# Intra-renal delivery of mesenchymal stem cells attenuates myocardial injury after reversal of hypertension in porcine renovascular disease

**DOI:** 10.1186/scrt541

**Published:** 2015-01-19

**Authors:** Alfonso Eirin, Xiang-Yang Zhu, Christopher M Ferguson, Scott M Riester, Andre J van Wijnen, Amir Lerman, Lilach O Lerman

**Affiliations:** Department of Internal Medicine, Division of Nephrology and Hypertension, Mayo Clinic, Rochester, MN USA; Department of Orthopedic Surgery, Mayo Clinic, Rochester, MN USA; Division of Cardiovascular Diseases, Mayo Clinic, Rochester, MN USA

## Abstract

**Introduction:**

Percutaneous transluminal renal angioplasty (PTRA) fails to fully improve cardiac injury and dysfunction in patients with renovascular hypertension (RVH). Mesenchymal stem cells (MSCs) restore renal function, but their potential for attenuating cardiac injury after reversal of RVH has not been explored. We hypothesized that replenishment of MSCs during PTRA would improve cardiac function and oxygenation, and decrease myocardial injury in porcine RVH.

**Methods:**

Pigs were studied after 16 weeks of RVH, RVH treated 4 weeks earlier with PTRA with or without adjunct intra-renal delivery of MSC (10^6 cells), and controls. Cardiac structure, function (fast-computed tomography (CT)), and myocardial oxygenation (Blood-Oxygen-Level-Dependent- magnetic resonance imaging) were assessed *in-vivo*. Myocardial microvascular density (micro-CT) and myocardial injury were evaluated *ex-vivo*. Kidney venous and systemic blood levels of inflammatory markers were measured and their renal release calculated.

**Results:**

PTRA normalized blood pressure, yet stenotic-kidney glomerular filtration rate, similarly blunted in RVH and RVH + PTRA, normalized only in PTRA + MSC-treated pigs. PTRA attenuated left ventricular remodeling, whereas myocardial oxygenation, subendocardial microvascular density, and diastolic function remained decreased in RVH + PTRA, but normalized in RVH + PTRA-MSC. Circulating isoprostane levels and renal release of inflammatory cytokines increased in RVH and RVH + PTRA, but normalized in RVH + PTRA-MSC, as did myocardial oxidative stress, inflammation, collagen deposition, and fibrosis.

**Conclusions:**

Intra-renal MSC delivery during PTRA preserved stenotic-kidney function, reduced systemic oxidative stress and inflammation, and thereby improved cardiac function, oxygenation, and myocardial injury four weeks after revascularization, suggesting a therapeutic potential for adjunctive MSC delivery to preserve cardiac function and structure after reversal of experimental RVH.

## Introduction

Renovascular hypertension (RVH) is a manifestation of atherosclerotic renovascular disease, which is associated with progressive renal dysfunction and increased cardiovascular morbidity and mortality [1]. Among hypertensive patients undergoing echocardiography, cardiac structure and diastolic function are more compromised in RVH than in essential hypertensive patients, and its coexistence with significant renal failure further aggravates cardiac structural and functional abnormalities [2].

Restoring blood flow to the kidney by percutaneous transluminal renal angioplasty (PTRA) has become a common approach among interventional cardiologists and radiologists [3]. However, the Cardiovascular Outcomes in Renal Atherosclerotic Lesions (CORAL) trial recently demonstrated that PTRA does not confer any additional benefit over medical therapy with respect to the prevention of hospitalization or death from myocardial infarction, congestive heart failure and other cardiovascular causes [4]. In agreement, we have shown in porcine RVH that treatment with PTRA alone normalizes blood pressure without substantial improvement of cardiac diastolic function [5], warranting the need for adjunctive therapies to preserve the RVH myocardium.

Among insults responsible for persistent myocardial injury after reversal of hypertension are inflammation and oxidative stress, which might mediate the crosstalk between the kidney and heart. We have previously shown in porcine RVH increased stenotic-kidney release of isoprostanes [6], which are potent coronary vasoconstrictors [7]. Furthermore, we have demonstrated that post-stenotic porcine and human kidneys release several inflammatory cytokines [8, 9] that may accelerate target organ injury. Importantly, despite successful restoration of vessel patency with PTRA, inflammatory cytokines and oxidative stress markers remain elevated and glomerular filtration rate (GFR) fails to recover in both porcine and human RVH [10, 11], underscoring the need for strategies tailored to ameliorate inflammation and oxidative stress.

Mesenchymal stem cells (MSC) are undifferentiated nonembryonic stem cells with the ability to migrate and transdifferentiate into distinct phenotypes. These cells can be isolated from many tissues including adipose tissue and possess potent immunomodulatory properties via their paracrine anti-inflammatory actions [12]. Accumulating evidence suggests that MSC can directly contribute to both renal and cardiac repair [13]. For example, in a rat model of ischemic heart failure, a single intramyocardial injection of bone marrow-derived MSC decreases infarct area and preserves the left ventricle (LV) ability to contract during ischemia [14]. Likewise, intramyocardial administration of adipose tissue-derived MSC improves functional capacity in rats with myocardial infarction [15], supporting the value of this approach to preserve cardiac function.

Our group has previously demonstrated that a single intrarenal delivery of autologous MSC restores post-stenotic kidney structure and function in non-revascularized RVH pigs [8, 16]. Furthermore, intra-renal delivery of allogeneic MSC in conjunction with PTRA restored renal function and decreased stenotic-kidney inflammation, oxidative stress and fibrosis in porcine RVH [17, 18]. However, a key question is whether this approach is capable of indirectly blunting cardiac injury and dysfunction after reversal of RVH. This study tested the hypothesis that replenishment of MSC as an adjunct to PTRA would attenuate systemic oxidative stress and inflammation, improving cardiac function and oxygenation, and decreasing myocardial injury in porcine RVH.

## Methods

With the approval of the Mayo Clinic Animal Care and Use Committee, 28 domestic female pigs were studied after 16 weeks of observation. At baseline, 7 animals started a normal diet (regular pig chow) and the other 21 a 2%-cholesterol/15%-lard diet (TD-93296, Harlan-Teklad, Madison, WI, USA) to induce atherosclerosis [19].

Six weeks later, animals were anesthetized with 0.25 g of intramuscular tiletamine hydrochloride/zolazepam hydrochloride (Telazol®, Animal Health, Fort Dodge, IA, USA) and 0.5 g of xylazine, and anesthesia maintained with 0.2 mg/kg/minute of intravenous ketamine and 0.03 mg/kg/min of xylazine. Animals on the normal diet underwent a sham procedure, while in the other 21 RVH was induced by placing a local-irritant coil in the main renal artery using fluoroscopy, as previously described [20]. In addition, a telemetry system (Data Sciences International, St. Paul, MN, USA) was implanted in the left femoral artery to obtain daily records of mean arterial pressure (MAP) for the 10 following weeks [21, 22].

Six weeks after induction of RVH, renal angiography was performed in all animals to assess the degree of stenosis. Seven normal and seven RVH pigs underwent a sham procedure, whereas the other fourteen were treated with PTRA with or without a single intra-renal infusion of allogeneic MSC. PTRA was performed under fluoroscopic guidance by inflating a 7 mm balloon catheter wrapped with a tantalum stent to 8 atm pressure in the proximal-middle section of the renal artery. Then, the balloon was deflated and removed, leading to full restoration of luminal patency [23, 24].

Four weeks after PTRA, pigs were similarly anesthetized and renal angiography repeated. Single-kidney GFR, renal blood flow (RBF), and cardiac function were assessed using multi-detector computer tomography (MDCT), while myocardial oxygenation was evaluated by blood oxygen level-dependent magnetic resonance imaging (BOLD-MRI). Inferior vena cava (IVC) samples were collected for isoprostane levels (enzyme immunoassay kit), plasma renin activity (PRA, GammaCoat PRA kit; DiaSorin, Inc., Stillwater, MN, USA), cholesterol panels and creatinine. IVC and stenotic-kidney renal vein (RV) levels of interleukin (IL)1α, IL1(receptor)rα, IL1β, IL10, e-selectin, and endothelin (ET)-1 were measured by luminex (Millipore, Billerica, MA, USA), and their gradient (RV-IVC) and net renal release (gradientxRBF) calculated [8, 9].

After a three-day recovery period, animals were euthanized with a lethal dose of intravenous sodium pentobarbital (100 mg/kg, Fatal Plus, Vortech Pharmaceuticals, Dearborn, MI, USA). The heart was removed, a segment of the LV prepared for micro-CT studies, and the remaining LV tissue was frozen in liquid nitrogen at −80°C or preserved in formalin for *in vitro* studies.

### Renal function

Stenotic-kidney RBF and GFR were measured using MDCT (Somatom Definition-64, Siemens Medical Solution, Forchheim, Germany), as previously described [25, 26]. Briefly, multiple consecutive scans were performed following a central venous injection of iopamidol (0.5 mL/kg per 2 seconds), and images reconstructed and displayed with the Analyze™ software package (Biomedical Imaging Resource, Mayo Clinic, Rochester, MN, USA). Data were analyzed by selecting regions of interest from cross-sectional images from the aorta, renal cortex, and medulla, which generates tissue attenuation curves [27]. RBF was calculated as the sum of the products of cortical and medullary perfusions and corresponding volumes, whereas GFR was assessed from the cortical curve using the slope of the proximal tubular curve.

### Cardiac function and oxygenation

Cardiac systolic and diastolic functions and LV muscle mass (LVMM) were measured using MDCT, as previously described [22, 28]. Images were analyzed with Analyze™. In brief, Early (E) and late (A) LV filling rate were measured from the positive slopes of volume/time curves and E/A ratio calculated using MATLAB® (MathWork, Natick, MA, USA) [29, 30]. Myocardial perfusion was calculated from time-attenuation curves obtained from the anterior cardiac wall before and during a five-minute intravenous infusion of adenosine (400 μg/kg/minute) [31]. Myocardial oxygenation was assessed using BOLD-MRI on a 3 T, Signa EchoSpeed (GE Medical Systems, Milwaukee, WI, USA) scanner, as previously described [5]. For MRI, animals were anesthetized with 1% to 2% isoflurane and scans performed during suspended respiration before and after 400 μg/kg/minute of intravenous adenosine. The relaxivity index R2*, which inversely correlates with tissue oxygenation, was calculated in each voxel by fitting the MR signal intensity versus echo times to a single exponential function. For data analysis, regions of interest were traced in the septum in each slice and images analyzed using MATLAB 7.10 (MathWorks), as previously described [32].

### MSC isolation, characterization, function, delivery and tracking

Porcine omental abdominal adipose tissue (5 to 10 g) was collected and allogeneic MSC isolated using a standard protocol [33]. In brief, cells were digested in collagenase-H for 45 minutes, filtered and cultured in endothelial cell growth media-2 for three weeks in 37°/5% CO2, and the third passage preserved in Gibco Cell Culture Freezing Medium (Life Technologies, Grand Island, NY, USA) at −80°C until transplantation. MSC were characterized by immunostaining and fluorescence-activated cell sorting analysis to determine cellular phenotype for the MSC markers CD44 (1:100; abcam, Cambridge, MA, USA) and CD90 (1:100; BD Pharmigen, San Jose, CA, USA). MSC characterization was confirmed by their trans-differentiation into osteocytes (mouse anti-human osteocalcin antibody and alizarin red staining), chondrocytes (goat anti-human aggrecan antibody) and adipocytes (goat anti-mouse FABP-4 antibody and oil red staining) (R&D Systems, Pittsburgh, PA, USA) [17].

MSC function was also tested [34, 35] in a different batch of MSC (isolated from three pigs) of the same passage, which had also been previously frozen for several weeks, thawed and recovered for 24 hours. MSC proliferative activity was determined in a plate reader at 490 nm by MTS assay (CellTiter 96 Non-Radioactive Cell Proliferation Assay; Promega, Madison, WI, USA), as previously described [35]. MSC migratory capacity was tested using a QCMTM Haptotaxis cell migration kit (Millipore) and read at 562 nm [34]. Finally, tube formation assay (BD Biosciences, Bedford, MA, USA) was performed to assess the ability of MSC to incorporate into vascular structures formed by human umbilical vein endothelial cells (HUVEC) in matrigel. MSC (1 × 10^4^) pre-labeled with DiI (Molecular Probes, Grand Island, NY, USA) were mixed and plated together with HUVEC (PromoCell, Heidelberg, Germany) (4 × 10^4^). Tube length and number were counted in random 20X fields and measured using ZEN®, 2012 blue edition (Carl ZEISS SMT, Oberkochen, Germany).

MSC were labeled with a fluorescent membrane dye (CM-DiI) and kept in 10 ml PBS (10^6^ cells/mL), and injected immediately after PTRA slowly through a balloon placed in the renal artery proximal to the stenosis. Four weeks after delivery, labeled MSC were tracked in frozen LV sections stained with 4′,6-diamidino-2-phenylindole (DAPI) nuclear stain and in stenotic-kidney sections stained with DAPI and the tubular marker cytokeratin (AbD Serotec, Raleigh, NC, USA). Stenotic-kidney and myocardium MSC retention rate (percentage of injected cells that remained in the organ) was calculated, as previously described [10, 17].

### Microvascular remodeling

Myocardial microvascular architecture was assessed using a micro-CT scanner. The proximal left anterior descending artery was cannulated and perfused under physiological pressure with an intravascular contrast agent (MV-122, Flow Tech, Carver, MA, USA). A transmural section of the LV (2 cm^3^) was scanned and spatial density of small (<200 μm), medium (200 to 300 μm) and large (>300 μm) microvessels in the sub-epicardium and sub-endocardium calculated [21, 36] using Analyze™. In addition, immunostaining with anti-α-smooth muscle actin (SMA) antibody (DakoCytomation, Glostrup, Denmark) was performed and media-to-lumen ratio calculated to assess microvascular wall thickening [22].

### Oxidative stress and inflammation

Myocardial oxidative stress was evaluated by the *in situ* production of superoxide anion, detected by fluorescence microscopy using dihydroethidium (DHE) [37]. Inflammation was evaluated in myocardial sections by double immunofluorescent staining for pro-inflammatory CD68+/inducible nitric oxide synthase (iNOS) + (M1) and reparative CD68+/Arinase-1 (M2) macrophages (1:100; Abnova Inc., Walnut, CA, USA) [5]. In addition, myocardial expression of IL-10 (1:200; Biotechnology, Santa Cruz, CA, USA) was determined by western blot and normalized for a GADPH loading control.

### Myocardial remodeling and fibrosis

Cross-sections (5-μm) of the LV were stained with H & E, Sirius red, and trichrome to assess myocyte cross-sectional area, interstitial collagen deposition and fibrosis, respectively. Slides (one per animal) were examined in a blinded manner using ZEN®, as previously described [5, 32].

### Statistical analysis

Statistical analysis was performed using JMP 9.0 (SAS Institute, Cary, NC, USA). Data were expressed as mean ± standard deviation for normally distributed variables or median (range) for non-Gaussian distributed data. Parametric (one-way analysis of variance (ANOVA) followed by unpaired Student’s t-test) and non-parametric (Wilcoxon followed by Kruskal-Wallis) tests were used when appropriate, and significance accepted for *P* <0.05.

## Results

### PTRA decreased blood pressure

**Figure 1:**
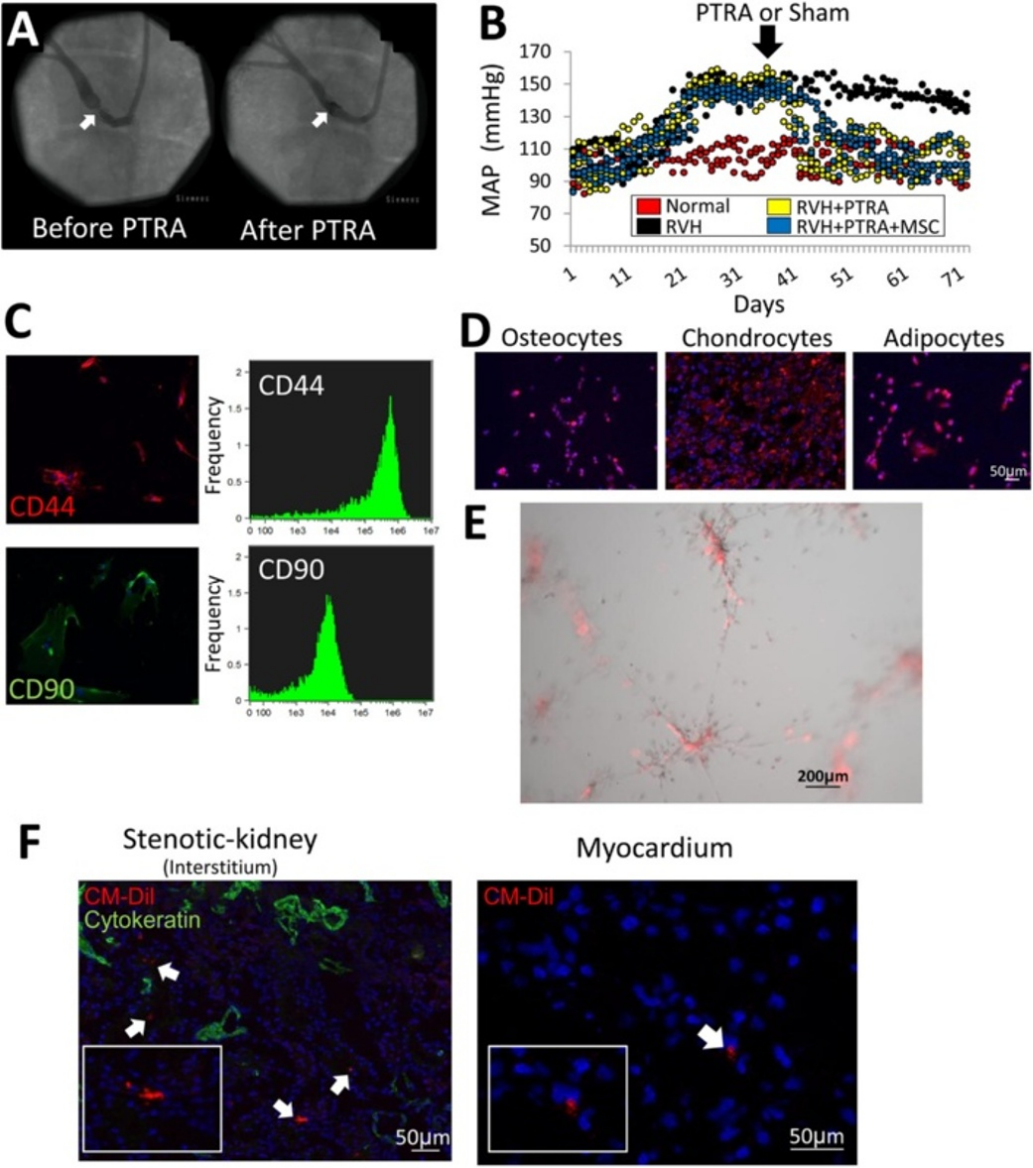
**Hemodynamic effect of renal artery stenosis and characteristics of MSC. A)** Renal angiography before and after PTRA, showing successful restoration of vessel patency (arrows). **B)** Mean arterial pressure (MAP) measured by telemetry decreased after PTRA. **C)** Immunostaining (left) and FACS (right) showing that adipose tissue-derived MSC expressed CD44 and CD90 surface markers. **D**) Representative immunostaining showing that MSC trans-differentiated into osteocytes, chondrocytes and adipocytes *in vitro*. **E)** DiI-labeled MSC (red) incorporated into tubes formed by HUVEC (grey). **F)** Representative fluorescence of CM-DiI-labeled MSC (red, arrows) and cytokeratin (green) in the stenotic-kidney (left, X20) and myocardium (right, X40) four weeks after administration. FACS, fluorescence activated cell sorting; HUVEC, human umbilical vein endothelial cells; MSC, mesenchymal stem cells; PTRA, percutaneous transluminal renal angioplasty.

Six weeks after stenosis induction, all RVH, RVH + PTRA and RVH + PTRA + MSC pigs achieved hemodynamically significant and comparable degrees of stenosis (80.5 ± 14.5%, 78.8 ± 10.0% and 78.6 ± 15.3%, respectively) (*P* = 0.96 ANOVA). Four weeks after successful PTRA (0% stenosis in all PTRA-treated pigs), MAP normalized in RVH + PTRA and RVH + PTRA + MSC pigs (Table 1, Figure 1A-B). Total cholesterol and low-density lipoprotein (LDL) levels were elevated in all RVH groups compared to normal, whereas high-density lipoprotein (HDL) and triglyceride levels did not differ among the groups (Table 1).

**Table 1:** **Systemic characteristics and cardiac function of study groups (n = 7 each) four weeks after PTRA or sham**

Parameter	NORMAL	RVH	RVH + PTRA + Vehicle	RVH + PTRA + MSC
Degree of stenosis (%)	0	78.3 ± 13.3*†	0	0
Body weight (Kg)	48.3 ± 1.0	51.3 ± 3.6	50.7 ± 3.9	54.1 ± 3.7
Mean blood pressure (mmHg)	97.8 ± 9.5	137.9 ± 5.5*†	99.0 ± 9.0	96.4 ± 2.6
Cholesterol (mg/dl): Total	92.2 ± 4.8	483.7 ± 34.8*	425.5 ± 14.4*	407.3 ± 26.8*
HDL	105.3 ± 33.2	159.0 ± 15.8	155.5 ± 13.1	144.6 ± 16.7
LDL	46.0 ± 11.3	341.5 ± 48.2*	279.2 ± 22.3*	250.5 ± 23.5*
Triglycerides (mg/dl)	7.5 (5–10)	6 (4–8)	5.5 (4–17)	8 (3–9)
Serum creatinine (mg/dl)	1.3 ± 0.04	1.9 ± 0.1*†	1.9 ± 0.1*†	1.4 ± 0.1
GFR (ml/min)	77.9 ± 3.9	52.2 ± 2.7*†	56.9 ± 3.2*†	72.3 ± 3.1
Plasma renin activity (ng/ml/hour)	0.2 (0.07-0.25)	0.19 (0.02-0.30)	0.21 (0.06-0.24)	0.13 (0.06-0.25)
Isoprostane (pg/ml)	109.1 ± 7.3	231.6 ± 38.3*†	212.1 ± 22.6*†	130.4 ± 11.7
Heart rate (bpm)	72.5 (63–104)	84.0 (69–123)	75.3 (70–96)	78.0 (66–119)
Stroke volume (ml)	40.8 ± 2.0	44.3 ± 1.7	43.0 ± 1.0	44.7 ± 3.4
Ejection fraction (%)	63.9 ± 2.4	59.7 ± 2.3	62.6 ± 2.2	65.1 ± 4.7
Cardiac output (L/min)	3.2 ± 0.3	3.6 ± 0.2	3.5 ± 0.2	3.4 ± 0.2
LVMM (g/kg body weight)	1.6 ± 0.1	2.4 ± 0.2*†	1.8 ± 0.1	1.8 ± 0.1
E/A ratio	1.2 ± 0.1	0.7 ± 0.1*†	0.8 ± 0.1*†	1.1 ± 0.1
EDV (ml)	84.3 ± 2.2	66.0 ± 2.7*†	65.6 ± 2.4*†	80.6 ± 4.5
Myocardial perfusion *(ml/min/g)*:				
*Baseline*	0.9 ± 0.1	0.7 ± 0.04*†	0.7 ± 0.04*†	0.8 ± 0.1
*Response to adenosine*	1.0 ± 0.1‡	0.7 ± 0.04*†	0.7 ± 0.1*†	0.9 ± 0.1‡

*P <0.05 versus normal, †*P* <0.05 versus RVH + PTRA + MSC, ‡P <0.05 versus baseline. Bpm, beats per minute; E/A early and late left ventricular filling ratio; EDV: end diastolic volume; GFR, glomerular filtration rate; HDL: high-density lipoprotein; LDL: low-density lipoprotein; LVMM: left ventricular muscle mass; MSC: mesenchymal stem cells; PTRA: percutaneous transluminal renal angioplasty; RVH: renovascular hypertension. Italics were used as subheadings of Myocardial perfusion

### MSC characterization, function and engraftment

Isolated and cultured MSC expressed CD44 and CD90, and transdifferentiated into osteocytes, chondrocytes and adipocytes (Figure 1C-D). Thawed MSC vigorously proliferated, migrated and formed tubes (Table 2, Figure 1E). Four weeks after intra-renal administration, 13% to 14% of injected MSC were detected at the renal tubulointerstitial compartment, yet only 0.03% to 0.05% of injected cells were found in the myocardium (Figure 1 F).

**Table 2:** **Function of porcine mesenchymal stem cells before delivery**

Parameter	
Proliferation (OD)	0.34 ± 0.01
Migration (OD)	2.49 ± 0.26
Tube Formation:	
Tube (number/field)	15.64 ± 2.32
Tube length (mm)	4.50 ± 1.05

### MSC improved renal function

Stenotic-kidney GFR was lower and serum creatinine levels higher in RVH and RVH + PTRA compared to normal, yet normalized in PTRA + MSC-treated pigs (Table 1, *P* <0.05 versus RVH and RVH + PTRA, *P* >0.05 versus normal). Systemic PRA was similar in all groups, as common in chronic RVH [38].

### MSC improved cardiac function and oxygenation

**Figure 2:**
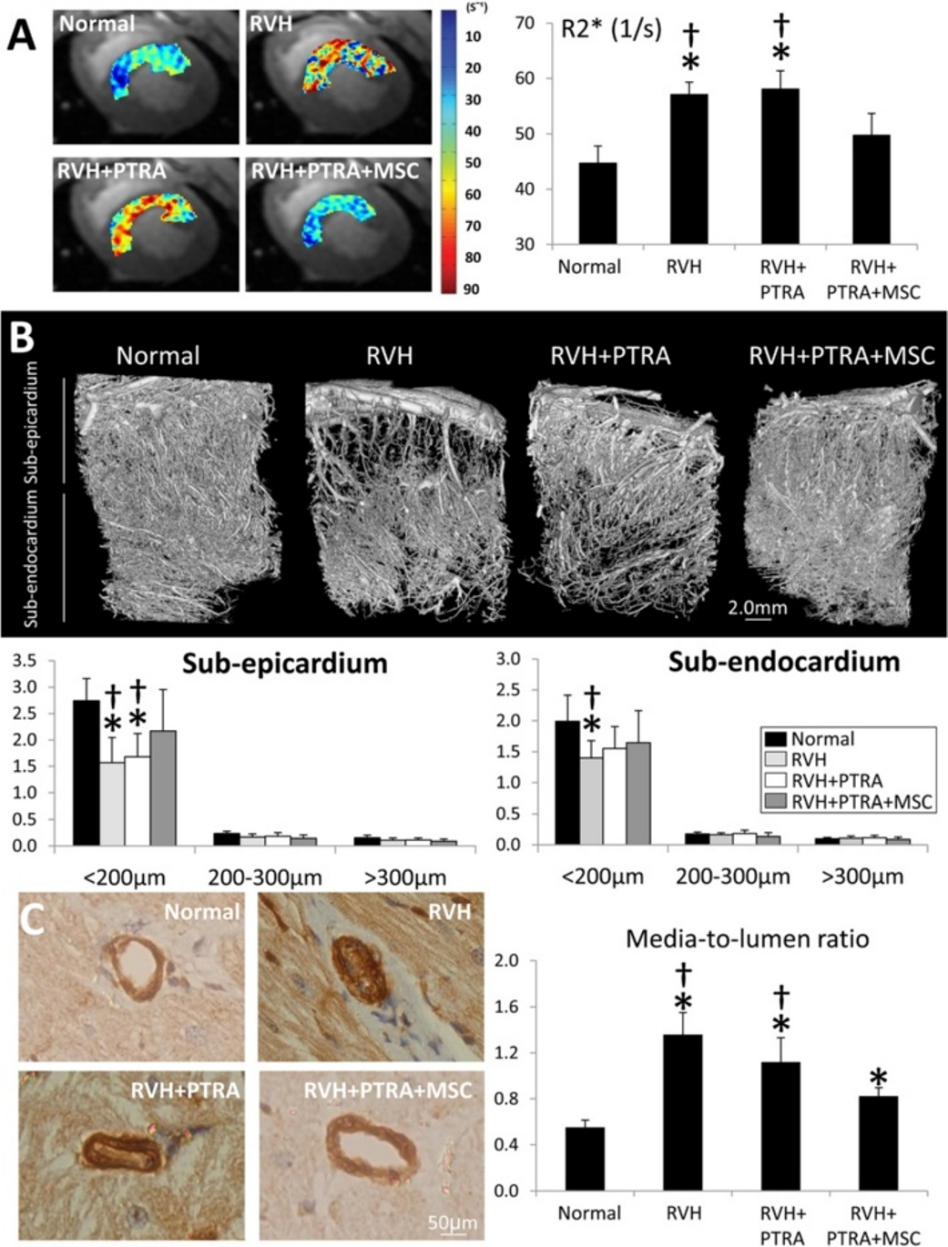
**MSC improved renal oxygenation and microvascular architecture. A)** Blood oxygen level-dependent (BOLD) MRI images of the LV (left), showing hypoxic myocardium (yellow-red) in RVH, and quantification of R2* index (right). *P <0.05 versus normal, †*P* <0.05 versus RVH + PTRA + MSC. **B)** Representative myocardial micro-CT images (top) and quantification of spatial density of small, medium and large microvessels in the subepicardium and subendocardium (bottom). **C)** Myocardium sections stained with α-smooth muscle actin (left) and quantification of vessel media-to-lumen ratio (right). *P <0.05 versus normal, †*P* <0.05 versus RVH + PTRA + MSC. CT, computed tomography; LV, left ventrical; MSC, mesenchymal stem cells; MRI, magnetic resonance imaging; PTRA, percutaneous transluminal renal angioplasty; RVH, renovascular hypertension.

Heart rate, stroke volume, ejection fraction and cardiac output were similar among the groups (Table 1, *P* >0.05, ANOVA). However, LVMM was higher in RVH compared with normal, but restored to normal levels in both PTRA-treated groups. E/A ratio and EDV were lower in RVH compared to normal, unchanged by PTRA, and normalized only in MSC-treated pigs. Both myocardial perfusion and its response to adenosine were blunted in RVH and RVH + PTRA, but normalized in RVH + PTRA + MSC pigs (Table 1). Likewise, R2* values were similarly elevated in RVH and RVH + PTRA compared to normal, but were restored to normal levels in RVH + PTRA + MSC (Figure 2A).

### Microvascular remodeling was attenuated in MSC-treated pigs

Sub-epicardial and sub-endocardial densities of medium and large microvessels were similar among the groups (Figure 2B). The number of small vessels in the sub-epicardium was reduced in RVH, but did not differ from normal levels in either PTRA-treated group. However, sub-endocardial density of small size vessels was equally decreased in RVH and RVH + PTRA compared to normal, but normalized in PTRA-MSC-treated pigs (Figure 2B). Media-to-lumen ratios were higher in all RVH compared to normal, but improved only in RVH + PTRA + MSC pigs (Figure 2C).

### MSC decreased inflammation and oxidative stress

**Figure 3:**
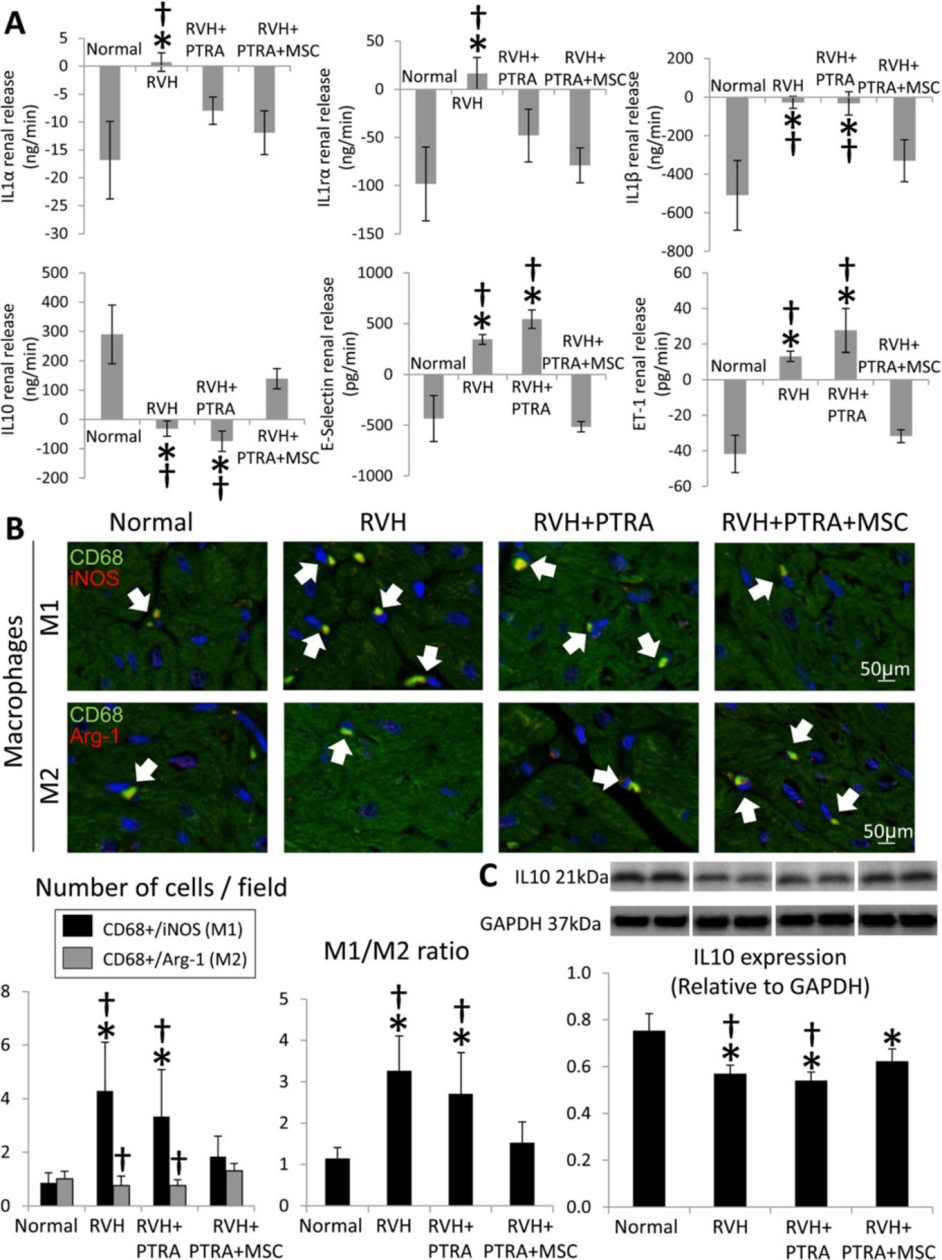
**MSC decreased inflammation. A)** Renal release of interleukin (IL)1α, IL1rα, IL1β, IL10, e-selectin, and endothelin (ET)-1 in study groups. **B)** Immunostaining for CD68/iNOS (M1) and CD68/Arginase-1 (M2) macrophages (top), their quantification and ratio (bottom). **C)** Representative immunoblots and myocardial protein expression of IL10 in normal, RVH, RVH + PTRA, and RVH + PTRA + MSC pigs. *P <0.05 versus normal, †*P* <0.05 versus RVH + PTRA + MSC. MSC, mesenchymal stem cells; PTRA, percutaneous transluminal renal angioplasty; RVH, renovascular hypertension.

Renal release of IL1α and IL1rα was elevated in RVH compared to normal, but normalized in both PTRA-treated groups (Figure 3A). However, release of IL-1β, e-selectin and ET-1 were similarly higher in RVH and RVH + PTRA compared to normal, but normalized in PTRA + MSC pigs. Renal release of IL10 was similarly decreased in RVH and RVH + PTRA, yet restored to normal levels in RVH + PTRA + MSC. The number of myocardial M1 macrophages was elevated in RVH and RVH + PTRA animals, but normalized in RVH + PTRA + MSC (Figure 3B). In contrast, the number of reparative M2 macrophages was elevated only in PTRA + MSC-treated pigs. Furthermore, the M1/M2 ratio was higher in RVH and RVH + PTRA compared to normal, but normalized in RVH + PTRA + MSC. Myocardial expression of IL-10 was downregulated in all RVH compared to normal, but improved only in PTRA + MSC-treated pigs (Figure 3C).

**Figure 4:**
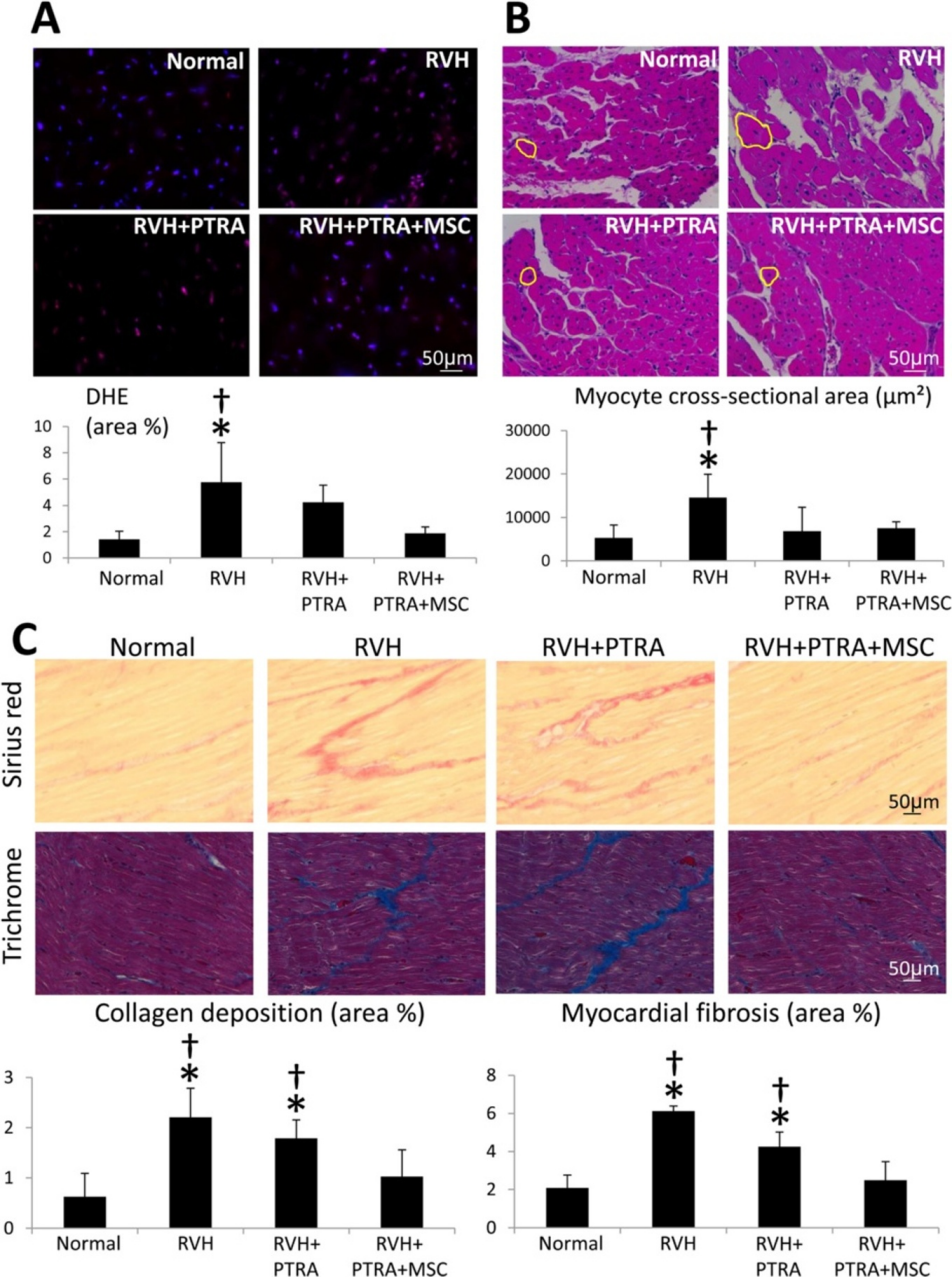
**MSC ameliorated myocardial oxidative stress, remodeling, and fibrosis. A)** Myocardial production of superoxide anion, detected by DHE (top), and its quantification (bottom). **B)** Left ventricular sections stained with H & E (top) and quantification of myocyte cross-sectional area (bottom). **C)** Representative staining and quantification of Sirius red and trichrome. *P <0.05 versus normal, †*P* <0.05 versus RVH + PTRA + MSC. DHE, dihydroethidium; MSC, mesenchymal stem cells; PTRA, percutaneous transluminal renal angioplasty; RVH, renovascular hypertension.

Circulating levels of isoprostanes were higher in RVH and RVH + PTRA compared to normal, but were restored to normal levels in RVH + PTRA + MSC (Table 1). Likewise, *in situ* production of superoxide anion was increased in RVH and RVH + PTRA, and normalized only in PTRA + MSC-treated piss (Figure 4A).

### MSC ameliorated myocardial remodeling and fibrosis

Myocyte cross-sectional area was higher in RVH compared to normal, but normalized in both PTRA-treated groups (Figure 4B). Both collagen deposition and fibrosis were elevated in RVH and RVH + PTRA compared to normal, yet normalized in PTRA + MSC-treated pigs (Figure 4C).

## Discussion

This study demonstrates that adjunctive MSC delivery in addition to PTRA decreased myocardial remodeling and improved cardiac function in porcine RVH, possibly by preserving stenotic-kidney function and decreasing renal release and systemic levels of noxious and vasoconstrictor humoral factors. These observations support MSC delivery during PTRA as a promising therapeutic intervention for preserving the myocardium in experimental RVH.

Several deleterious pathways may account for persistent cardiac injury after reversal of RVH. We have previously shown in porcine non-atherosclerotic RVH that PTRA improved coronary microvascular function and architecture and reversed myocardial hypertrophy and diastolic dysfunction [39]. However, superimposition of atherosclerosis exacerbates the effect of RVH on the myocardial microvasculature, which may compromise cardiac outcomes after revascularization [28]. Indeed, coronary artery disease in patients with RVH is a predictor of worse outcomes after renal revascularization, likely reflecting diffuse atherosclerotic disease [40].

Inflammation and oxidative stress are also important determinants of persistent cardiac dysfunction after renal revascularization. Endothelial activation, an early event in atherosclerosis, is characterized by increased plasma concentration of soluble adhesion molecules such as e-selectin that mediate adhesion of circulating leukocytes to the vascular wall, contributing to cardiac injury and dysfunction [41]. Endothelial activation is associated with increased release of the potent vasoconstrictor ET-1 [42]. Importantly, ET-1 and IL-1 have been implicated in the pathophysiology of LV hypertrophy and myocardial dysfunction [43, 44]. Similarly, reactive oxygen species (ROS) levels are increased in the myocardium of 2 kidney-1 clip (2K1C) rats, implicating oxidative stress in RVH-induced cardiac hypertrophy [45]. Importantly, in pigs with RVH, myocardial inflammation and oxidative stress persists after PTRA alone, associated with myocardial remodeling and impaired diastolic function [5].

We have previously shown that post-stenotic porcine and human kidneys release several inflammatory cytokines that accelerate renal injury [8, 9] and might damage the remote myocardium. We have also shown in porcine RVH that selective improvement of renal function reduces circulating levels of these noxious mediators, decreasing myocardial fibrosis and enhancing microvascular integrity, architecture and cardiac diastolic function [6].

Substantial evidence suggests that MSC can contribute to both renal and cardiac repair [13]. We have previously shown in porcine RVH that intra-renal delivery of MSC during PTRA preserves renal structure and function distal to a stenosis [17, 18]. Moreover, MSC decreased release of inflammatory cytokines and improved renal function in porcine non-revascularized kidneys [8]. Our study extends these observations, demonstrating decreased release of pro-inflammatory markers accompanied by normalized release of the anti-inflammatory IL-10 induced by MSC in PTRA-treated pigs. Consequently, an anti-inflammatory effect of MSC was conferred on the heart, reflected in increased expression of IL-10 and decreased pro-inflammatory/reparative macrophage ratio in RVH + PTRA + MSC pigs. Furthermore, renal release of e-selectin and ET-1 were normalized in PTRA-MSC treated pigs, suggesting decreased systemic endothelial activation and vasoconstrictor activity, which might have improved myocardial perfusion responses.

Additionally, we found that treatment with PTRA + MSC normalized systemic levels of the oxidative stress marker and potent coronary vasoconstrictor isoprostane and decreased myocardial production of superoxide anion. This might be secondary to their anti-inflammatory effect, which might have, in turn, blunted oxidative stress. For example, interferon-γ triggers formation and release of ROS in cardiovascular disease [46] and tumor necrosis factor-α increases ROS generation in the rat myocardium [47]. Likewise, IL-1 pretreatment of isolated rat hearts causes polymorphonuclear leukocyte accumulation associated with increased hydrogen peroxide-dependent oxidative stress, suggesting a direct link between myocardial inflammation and oxidative stress [48]. Oxidative stress also contributes to RVH-induced myocardial microvascular remodeling, leading to impaired perfusion [30]. In agreement, we found a decreased number of small microvessels in the sub-epicardium and sub-endocardium in RVH pigs. Although the number of small sub-epicardial microvessels was similarly restored in both PTRA-treated groups, the sub-endocardial density of small microvessels was normalized only in RVH + PTRA + MSC, accompanied by a reduced media-to-lumen ratio. MSC-induced protection of microvascular architecture and decreased vessel remodeling might have improved blood supply and oxygen delivery to the myocardium, disclosed by normalized R2* index.

Notably, myocardial collagen deposition and fibrosis, similarly upregulated in RVH and RVH + PTRA, were restored to normal levels only in PTRA + MSC-treated pigs. Although PTRA prevented LV remodeling (LVMM and myocyte cross-sectional area), diastolic function (E/A ratio and EDV) normalized only in PTRA + MSC-treated pigs, possibly mediated by decreased oxidative stress, inflammation and fibrosis. In line with these observations, myocardial perfusion and its response to adenosine normalized exclusively in RVH + PTRA + MSC, possibly secondary to restoration of microvascular structure and function and downregulated renal release and systemic levels of the potent vasoconstrictors ET-1 and isoprostane.

MSC renoprotection is likely attributable to their capacity to secrete paracrine factors rather than their ability to engraft the kidney. We have previously shown that MSC are mostly observed at the interstitium four weeks after injection, some incorporate into proximal renal tubules [16], and very few engraft in blood vessels [17]. However, few of the engrafted cells showed trans-differentiation to renal cells, suggesting that the main effect of MSC in the kidney is exerted by paracrine actions. We have also previously shown that porcine MSC actively secrete the potent pro-angiogenic mediator vascular endothelial growth factor in conditioned medium *in vitro*[16, 17]. Likewise, porcine MSC co-cultured with monocytes induce a phenotypic switch of pro-inflammatory to reparative macrophages, suggesting a direct paracrine anti-inflammatory effect [8]. Furthermore, we have recently demonstrated that porcine MSC release extracellular vesicles that possess an important set of transcription factors, which might be able to reprogram target cells or otherwise modify their biological phenotype [49]. Therefore, paracrine actions of functionally active cells might have contributed to preserve the stenotic-kidney parenchyma. However, very few MSC were retained in the myocardium four weeks after delivery, arguing against a major contribution of direct MSC effects to attenuation of cardiac injury. The effects on the heart were likely achieved indirectly by the decrease in preponderance of systemic inflammatory, pro-oxidant and vasoconstrictor mediators.

In the current study, we used allogeneic MSC to simulate the use of existing ‘off-the-shelf’ MSC products, which allow generation of a large amount of cells from a small number of donors in a short period of time. Although T-cell recognition by the recipient of alloantigen may occur after injection of allogeneic cells, MSC are considered immune-privileged because of the lack of expression of costimulatory molecules [50]. Indeed, histological analysis showed no evidence of cellular rejection (for example, CD3 clusters) in tissue sections from RVH + PTRA + MSC pigs. Moreover, the current study demonstrates that post-thawing injection-ready MSC retain vital activities including proliferation, migration and angiogenic function (tube formation).

## Limitations

Study limitations include the use of young animals, short duration of the disease and lack of co-morbidities, such as essential hypertension, which may exacerbate RVH-induced cardiac damage. Nevertheless, cardiac injury and dysfunction in our porcine model closely resemble that in human RVH hearts. In our model, PTRA was more successful in decreasing blood pressure than typically seen in humans, yet myocardial injury and dysfunction persisted after revascularization. The use of enzyme immunoassay kit to measure isoprostane levels and E/A (rather than E/E’ ratio) to characterize LV dysfunction are also suboptimal. Future studies in human RVH are needed to validate these results and determine the optimal timing and dose of MSC.

## Conclusions

The current study showed that a single intra-renal infusion of allogeneic adipose tissue-derived MSC during PTRA indirectly improved cardiac function and oxygenation, and decreased myocardial injury four weeks after revascularization. MSC cardio-protective properties appear to be mediated by preservation of stenotic-kidney function, as well as attenuation of endothelial activation, vasoconstrictor activity, oxidative stress and inflammatory signals released from the ischemic kidney. These observations support MSC-based approaches as an adjunct therapy to preserve cardiac function and structure after PTRA in experimental RVH.

## Abbreviations

2K1C: 2 kidney-1 clip
A: late LV filling
BOLD-MRI: blood oxygen level-dependent MRI
ANOVA: analysis of variance
CORAL: Cardiovascular Outcomes in Renal Athjerosclerotic Lesions
DAPI: 4′,6-diamidino-2-phenylindole
DHE: dihydroethidium
E: early LV filling
EDV: end diastolic volume
ET: endothelin
GFR: glomerular filtration rate
H & E: hematoxylin and eosin
HDL: high-density lipoprotein
HUVEC: human umbilical vein endothelial cells
IL: interleukin
iNOS: inducible nitric oxide synthase
IVC: inferior vena cava
LDL: low-density lipoprotein
LV: left ventricle
LVMM: LV muscle mass
MAP: mean arterial pressure
MDCT: multi-detector computed tomography
MSC: mesenchymal stem cells
PBS: phosphate-buffered saline
PRA: plasma renin activity
PTRA: percutaneous transluminal renal angiopasty
r: receptor
RBF: renal blood flow
ROS: reactive oxygen species
RV: renal vein
RVH: renovascular hypertension
SMA: smooth muscle actin.
